# Dynamic observation of SARS‐CoV‐2 IgM, IgG, and neutralizing antibodies in the development of population immunity through COVID‐19 vaccination

**DOI:** 10.1002/jcla.24325

**Published:** 2022-03-02

**Authors:** Ruiwei Jiang, Xiaowen Dou, Min Li, Enyun Wang, Jiwen Hu, Dan Xiong, Xiuming Zhang

**Affiliations:** ^1^ 91594 School of Medicine Anhui University of Science and Technology Anhui China; ^2^ Medical Laboratory of Shenzhen Luohu Hospital Group Shenzhen Luohu People’s Hospital Shenzhen China; ^3^ Medical Laboratory of the Third Affiliated Hospital of Shenzhen University Shenzhen China

**Keywords:** COVID‐19 convalescent, COVID‐19 vaccine, immune response, neutralizing antibody, seropositivity

## Abstract

**Background:**

Currently, mass vaccine inoculation against coronavirus disease‐2019 (COVID‐19) has been being implemented globally. Rapid and the large‐scale detection of serum neutralizing antibodies (NAbs) laid a foundation for assessing the immune response against SARS‐CoV‐2 infection and vaccine. Additional assessments include the duration of antibodies and the optimal time for a heightened immune response.

**Methods:**

The performance of five surrogate NAbs—three chemiluminescent immunoassay (CLIA) and two enzyme‐linked immunosorbent assays (ELISAs)—and specific IgM and IgG assays were compared using COVID‐19‐vaccinated serum (*n* = 164). Conventional virus neutralization test (cVNT) was used as a criterion and the diagnostic agreement and correlation of the five assays were evaluated. We studied the antibody responses after the two‐dose vaccine in volunteers up to 6 months.

**Results:**

The sensitivity and specificity of five surrogate NAb assays ranged from 84% to 100%. Our cVNT results indicated great consistency with the surrogate assays. At 28 days after primary vaccination, the seropositivities of the NAbs, IgG, and IgM were 6%, 4%, and 13%, respectively. After the booster dose, seropositivities reached 14%, 65%, and 97%, respectively. Six months after receipt of the second dose, the NAb positive rate was eventually maintained at 66%. In all COVID‐19 convalescents, patients were detected with 100% NAb sat three months after discharge.

**Conclusion:**

COVID‐19 vaccine induced a humoral immune response lasting at least six months. Rapid serological detection was used as a proxy for identifying changes in immunity levels and as a guide to whether an individual may require a booster vaccination.

## 1 INTRODUCTION

The global pandemic of severe acute respiratory syndrome coronavirus 2 (SARS‐CoV‐2) has been ongoing since 2019, resulting in more than 416 million cases and 5.8 million deaths as of February 17, 2022.^1^ Ever‐emerging variants like B.1.617.2 that come with a greater ability to disseminate have the chance to prolong the pandemic or even worsen it. The global consensus that developing resistance to coronavirus disease‐2019 (COVID‐19) is needed has resulted in the acknowledgment that large‐scale, global population immunity urgently needed to provide protective immunity and interrupt COVID‐19’s transmission.^2^, ^3^, ^4^

Neutralizing antibodies (NAbs) develop from both natural infection and vaccination. Critically, they play major roles in protection against SARS‐CoV‐2 reinfection, prevention of pneumonia progression, and reduction of overall mortality.^5^, ^6^ Effective vaccination induces B cell responses to produce specific NAbs, which then competitively bind angiotensin‐converting enzyme 2 (ACE2) with receptor‐binding domain (RBD). Ultimately, this prevents the virus from binding to cellular receptors and entering into cells, thereby preventing infection. NAbs based on S protein detection are an important potential target for the sera of COVID‐19 vaccination participants and those who have been exposed to the disease. Previous studies have suggested that the neutralization titer of convalescent serum was approximately the same as that obtained from a vaccinated population.^7^

In view of the urgency posed by the COVID‐19 pandemic, 143 SARS‐CoV‐2 vaccines have begun clinical trials, with more than 195 additional vaccine candidates in either pre‐clinical or candidate phases.^8^ The COVID‐19 vaccines included the types of inactivated virus, weakened virus, and mRNA vaccines.^9^ 61.9% of the world population has received at least one dose of a COVID‐19 vaccine.^10^ Although vaccine‐elicited immunity has been reported in clinical trials, how long Nabs remain responsive in mass vaccination campaigns remains unknown. Given this, there is great need for the development of a rapid strategy for NAb detection in vaccine‐protected populations.

NAbs mostly rely on assays using a conventional virus neutralization test (cVNT) and pseudovirus‐based virus neutralization test (pVNT). Historically, cVNT has been used as a gold standard for protective NAb assays, but requires strict bio‐safety level (BSL‐3) facilities and the use of live virus entails an infection risk. Conversely, the alternative pVNT is time‐consuming and costly.^11^, ^12^ The critical conditions required for either cVNT or pVNT have greatly limited their use in large‐scale Nab screening for the evaluation of population immunity. Surrogate rapid tests have been developed for Nab monitoring, which have included enzyme‐linked immunosorbent assay (ELISA), chemiluminescent immunoassay (CLIA), and lateral flow immunoassays (LFAs).^13^ Previous work regarding the above has focused on the clinical performance of commercially available SARS‐COV‐2 serological kits in infectious and recovered COVID‐19 individuals; however, long‐term monitoring of the immune response in vaccinated cohorts by surrogate NAb assays has remained limited. Although SARS‐COV‐2 IgM and IgG are commonly used to diagnose SARS‐CoV‐2 infection, the specific antibody response in response to the effectiveness of the vaccine remains seldom reported.

In this study, we assessed performance of five surrogate NAb assays and specific antibody tests using cVNT. The characteristics of these assays on the NAb and specific antibodies were then compared. Finally, the dynamics of antibody titers in the vaccinated cohort was monitored using the surrogate assays during a six‐month observation period, which sought to evaluate the humoral immune response to the vaccination.

## 2 MATERIALS AND METHODS

### 2.1 Participants and sample preparation

In this long‐term study, a total of 164 healthy individuals were from the Shenzhen Luohu People's Hospital between November 2020 and July 2021. Participants received two injections of an inactivated SARS‐CoV‐2 vaccine (BBIBP‐CorV) in the deltoid muscle, with injections being 28 days apart. In total, 46 serum samples were obtained from 10 COVID‐19 convalescent patients who had continuous negative RNA results confirmed by reverse transcription–polymerase chain reaction (RT‐PCR) tests.

Baseline characteristics of participants are shown in Table S1. Briefly, the proportion of male and female participants was 46% and 54%, respectively, with an average age of 42.6 years old (range of 20–85 years old). Among 164 healthy individuals, 59 cases received a six‐month follow‐up for serological analysis; this included pre‐vaccine assessment to six‐month post‐vaccine. Patients who had recovered from COVID‐19 were discharged from the hospital and were visited with a three‐month observation after the onset of symptoms. At the time of the first inoculation, blood samples were taken from each volunteer at 0, 14, and 28 days post‐vaccination. After a four‐week interval, the second vaccination was administered and blood samples were collected after another 7, 14, 28, 90, and 180 days.

Except for the group receiving continuous monitoring, a total of 164 and 122 donors who were 14 days and 28 days, respectively, after completing the vaccination procedure were included for the performance comparison of different antibody assays. Briefly, blood was centrifuged at 3500g for 10 min (Allegra X‐15R, Beckman Coulter, USA), and the resulting serum was stored at −80°C prior to all serological tests. The study was authorized by the Ethics Committee of Shenzhen Luohu People's Hospital (2020‐LHQRMYY‐KYLL‐033). All participants gave their written informed consent for participation in this study.

### 2.2 Surrogate neutralizing and binding antibodies assays

#### 2.2.1 Chemiluminescent immunoassay (CLIA)

Surrogate Nabs assays were performed on three automatic CLIA devices include Axceed 260 (Bioscience Diagnostic Technology Co, Ltd., Tianjin China), iFlash3000 (YHLO Biotechnology Co., Ltd., Shenzhen China), and ECL8000 (Pulman Technology Co., Ltd. Shenzhen, China), respectively. The matched Nabs kits were provided by the manufacturers and the detection was strictly according to the instructions. Besides for NAbs, SARS‐CoV‐2 IgM and IgG were detected by Axceed kits. The characteristics of serological testing kits were offered in Table 1.

**TABLE 1:** Characteristics of rapid test kits for NAbs and SARS‐Cov‐2 IgM, IgG antibodies

Manufacturer	Assay name	Target antigen	Platform	Manufacturer's interpretation
Bioscience Diagnostic Technology, Tianjin	SARS‐CoV‐2 IgM Antibody kit	S1 protein (RBD)	Axceed 260 (CLIA)	Negative: S/CO < 1; Positive: S/CO ≥ 1
Bioscience Diagnostic Technology, Tianjin	SARS‐CoV‐2 IgG Antibody kit	S1 protein (RBD)	Axceed 260 (CLIA)	Negative: S/CO < 1; Positive: S/CO ≥ 1
Bioscience Diagnostic Technology, Tianjin	SARS‐CoV‐2 NAbs kit	S1 protein (RBD)	Axceed 260 (CLIA)	Negative: AU/ml < 2; Positive: AU/ml ≥ 2
YHLO Biotech, Shenzhen	SARS‐CoV‐2 NAbs kit	S1 protein (RBD)	iFlash3000 (CLIA)	Negative: AU/ml < 10; Positive: AU/ml ≥ 10
Lifotronic, Shenzhen	SARS‐CoV‐2 NAbs kit	S1 protein (RBD)	Ecl8000 (ECLIA)	Negative: AU/ml < 10; Positive: AU/ml ≥ 10
GenScript USA, Inc^a^	cPass™ SARS‐CoV‐2 NAbs ELISA Kit	S1 protein (RBD)	Manual ELISA	Negative: Inhibition < 30%; Positive: Inhibition ≥ 30%
Yaneng BIO science Shenzhen	Anti‐SARS‐CoV‐2 NAbs ELISA Kit	S1 protein (RBD)	Manual ELISA	Negative: Inhibition < 20%; Positive: Inhibition ≥ 20%

^a^ This kit had received emergency use authorization by the US Food and Drug Administration prior to October 19, 2020.

#### 2.2.2 Enzyme linked immunosorbent assay (ELISA)

Nabs in Yaneng and cPass kits were detected by ELISA method. cPass (GenScript) authorized by US Food and Drug Administration was applied as a referenced rapid NAbs kit. As the instruction, the serum (10 μl) was first diluted with buffer by nine times. Then, a mixture of HRP‐RBD solution and diluted serum (1:1, v/v) was added into the capture plates (coated with hACE2 receptor), and incubated. TMB substrate was used to produce the optical density (OD) at 450 nm. The control group without sample was prepared in parallel. Finally, the OD inhibition was calculated by the formula: OD Inhibition (%) = (1‐OD_sample_/OD_control_) × 100%. According to the manufacture, the value with OD inhibition above 30% was interpretated as negative result. The operation of Yaneng ELISA kits was similar to cPass, except for the positive cutoff value with OD Inhibition more than 20%.

### 2.3 Conventional virus neutralization test (cVNT)

Conventional VNT test used as a neutralization reference method was performed with 75 samples (14–28 days post‐vaccination). The detailed operation was as following. VeroE6 cells were cultivated at 37°C with 5% CO_2_ to grow to a monolayer of cells, and the test sera were inactivated at 56°C water bath for 30 min. In the biosafety cabinet, the serum sample was diluted with DMEM containing 2% fetal calf serum at the dilutions of 1:20, 1:40, 1:80, and 1:160, respectively. The equal amount of diluted sera and SARS‐CoV‐2 strain were incubated at 37°C for 1 h. The virus–sera mixtures were subsequently added to VeroE6 cells in culture plate, the virus titer was about 75 pfu per well. Then the supernatant solution was discarded, DMEM containing 0.8% sodium carboxymethyl cellulose was added and cultured at 37°C with 5% CO_2_ for 72 h. When plaque developed, the cells were fixed with 10% formaldehyde solution and stained by 0.5% crystal violet for a plaque‐inhibition test. Karber method was applied from the volume of serum and virus at the endpoint of the median tissue culture infectious dose (TCID50), and the titer above 1:20 was identified as NAbs generated in the individuals.

### 2.4 Statistical analysis

GraphPad Prism v9.0.0 and SPSS25.0 were used to perform the statistical analysis and mapping. Sensitivity and specificity were calculated with clinical negative cases and positive cases confirmed by cVNT, expressed with ratio and 95% CI. Spearman correlation was used to analyze the correlations of various kits with regard to antibodies titer. The area under the ROC curve (AUC) was estimated to assess the performance of serological assays to detect the presence of IgM, IgG, and NAbs. Wilcoxon rank‐sum test was used to determine the diagnostic differences among paired samples, and Cohen Kappa statistic was used to calculate the diagnostic agreement among groups. In view of antibody levels measured in different units by ELISA, CLIA, and cVNT, standardized data processing was carried out with each cutoff value. *p* < 0.05 was considered statistically significant.

## 3 RESULTS

### 3.1 Diagnostic accuracy of five surrogate neutralization assays compared with cVNT

**FIGURE 1:**
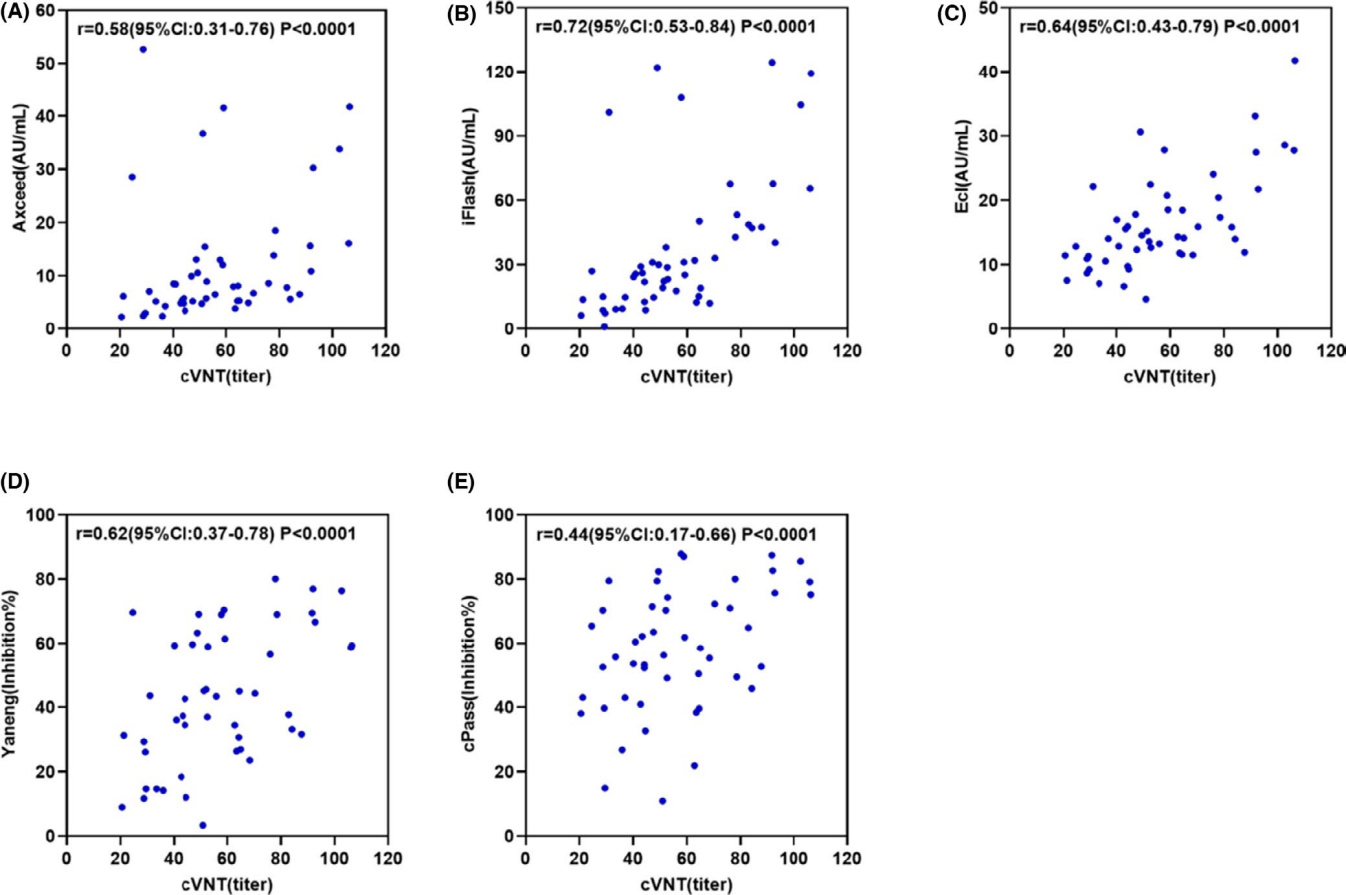
Spearman’ correlation analysis on neutralizing antibody titer between cVNT and each surrogate serology assay (*n* = 50)

As a standard reference, cVNT was conducted and 50 NAb‐positive samples were identified (Figure 1). The sensitivity and specificity were determined with NAb‐and NAb‐negative samples for the five rapid neutralization assays (Table 2). Results indicated that many types of kit were found with good specificity, with a sensitivity ranging from 84% to 100%. The best diagnostic performance was obtained with the Axceed NAb test. The diagnostic consistency (Cohen's Kappa above 0.78) with cVNTs was as follows: Axceed, cPass, iFlash, Ecl, and Yaneng. Using the manufacture's cutoff and NAb titers ≥ 1:20 as our criteria, the AUC of the empirical ROC was assessed for NAb, SARS‐CoV‐2 IgM, and IgG using the Axceed kits (Figure S1). Diagnostic capacity was obtained for IgM, IgG, and NAb with AUCs of 0.92 (95% CI; 0.86–0.97), 0.99 (0.99–1.00), and 1.00 (1.00–1.00), respectively. The group at 14 days post‐vaccine (*n* = 164) was assessed with IgM, IgG, and five NAb assays. Their resulting seropositivity was 41% for IgM, 88% for IgG, and 92%, 86%, 84%, 82%, and 78% for NAbs using the Axceed, iFlash, Yaneng, cPass, and Ecl tests, respectively.

**TABLE 2:** Diagnostic accuracy of various surrogate immunoassays to detect NAbs

Serological assays	Sensitively	Specificity	Cohen's Kappa
Axceed	100% (95CI:91.1%–100%)	100% (95CI:83.4%–100%)	1.00 (95CI:1.00–1.00) *p* < 0.0001
iFlash	86.0% (95CI:72.6%–93.7%)	100% (95CI:83.4%–100%)	0.80 (95CI:0.66–0.92) *p* < 0.0001
Ecl	84.0% (95CI:72.3%–92.4%)	100% (95CI:83.4%−100%)	0.78 (95CI:0.62–0.91) *p* < 0.0001
Yaneng	84.0% (95CI:72.3%–92.4%)	100% (95CI:83.4%–100%)	0.78 (95CI:0.63–0.91) *p* < 0.0001
cPass	92.0% (95CI:79.9%–97.4%)	100% (95CI:83.4%–100%)	0.89 (95CI:0.77–0.97) *p* < 0.0001

### 3.2 Performance comparison of four surrogate assays with cPass

**FIGURE 2:**
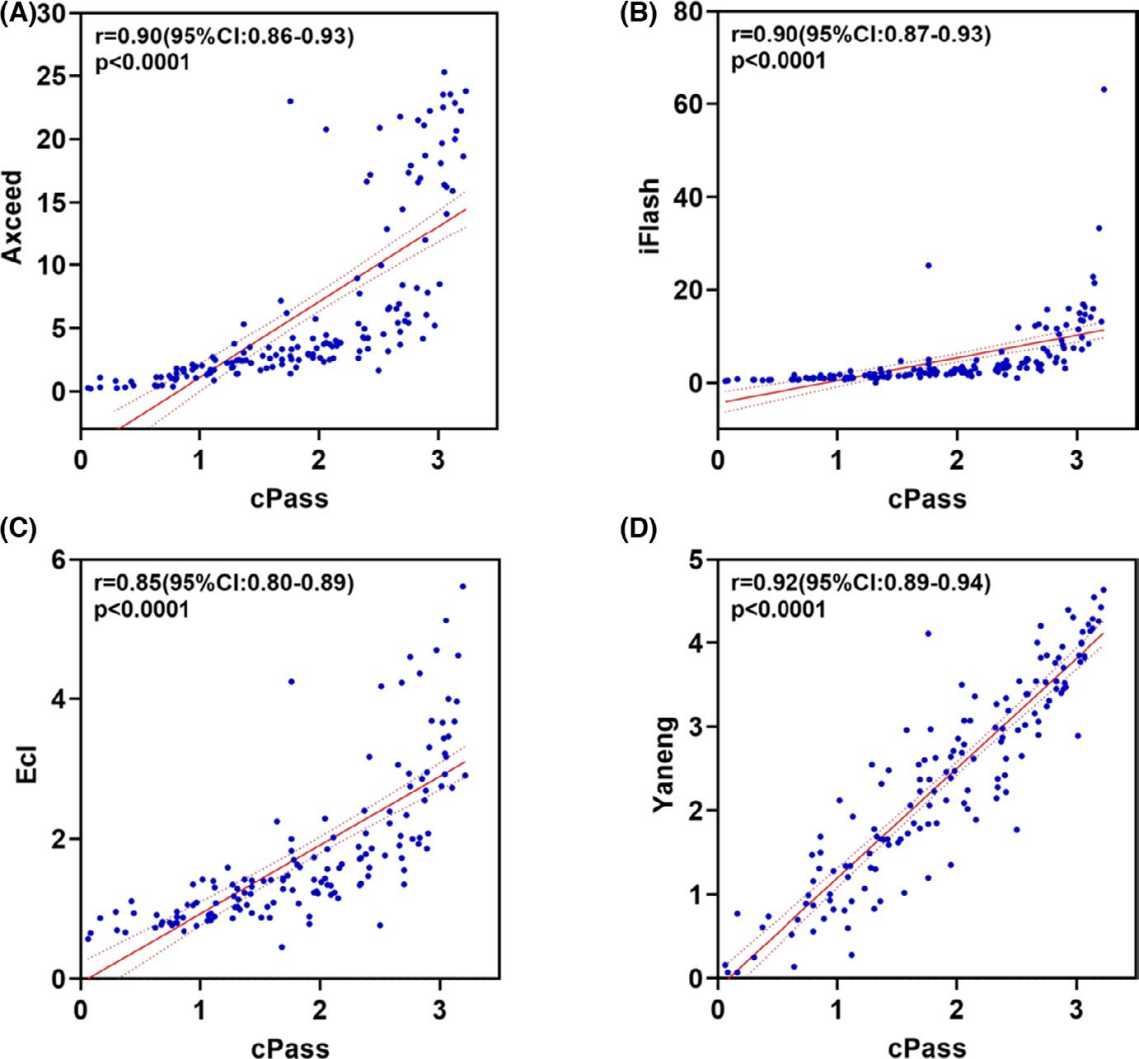
Spearman correlation analysis on NAb levels in serum post‐vaccination by cPass and four surrogate assays (*n* = 164). In view of measured in different units by ELISA and CLIA, standardized data processing was carried out with each cutoff value

Using the rapid kit‐cPass as a reference, the characteristics of the four surrogate assays were compared. Those that had good consistency with cPass were iFlash and Yaneng (kappa, 0.70), followed by Axceed and Ecl (kappa, 0.55). The titer correlation of 164 serum samples post‐immunization using cPass and each surrogate assay are shown in Figure 2. Close approximation with the Nab titer was observed with cPass and Yaneng, followed by the CLIA surrogate assays. Among the three CILA assays, there was moderate correlation with Axceed versus iFlash (*r*, .85), Axceed versus Ecl (*r*, .79), and iFlash versus Ecl (*r*, .92) (Figure S2).

### 3.3 Dynamic monitoring of SARS‐CoV‐2 IgM, IgG, and NAb in COVID‐19 vaccine participants

**FIGURE 3:**
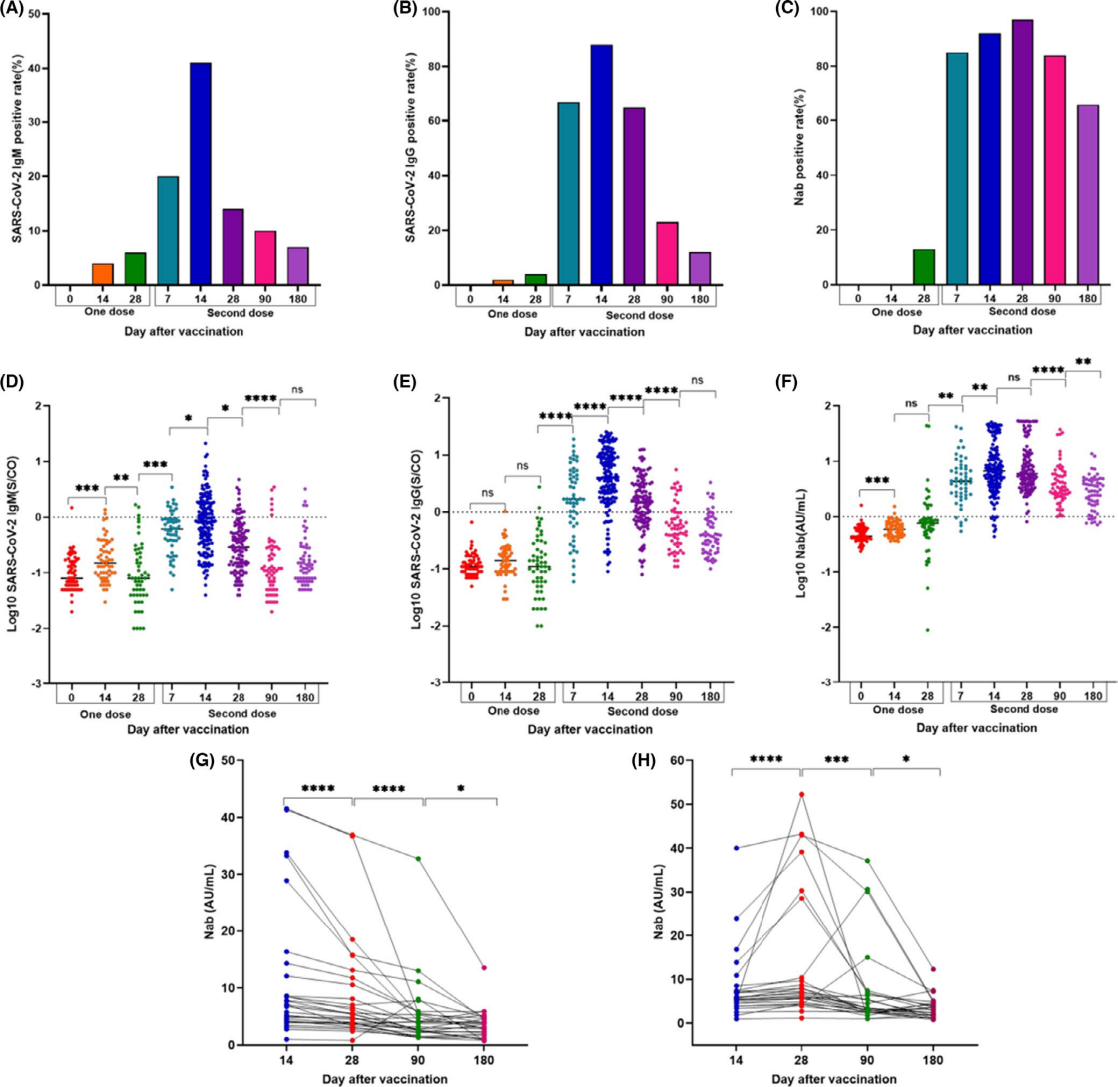
Seropositive of SARS‐CoV‐2 IgM, IgG, and neutralization antibody over vaccination time (A–C); the Changes in antibody levels were monitored with Axceed assay after COVID‐19 vaccination (D–H). Statistical analysis was carried out with paired parametric two‐tailed *t* test (ns: nondifferential; *, **, *** and **** indicates *p* < 0.05, <0.01, <0.001, and <0.0001, respectively). NAb levels changed at 14, 28, 90, and 180 days after the second dose of vaccine in 55 of the participants was analyzed by a paired Wilcoxon test (*p* < 0.05)

The seroconversion for SARS‐CoV‐2 IgM, IgG, and NAb were obtained from before inoculation to three months after the vaccine using the Axceed assay (Figure 3A,C). Both IgM (4%) and IgG (2%) was first detected with seropositivity at two‐weeks after the first dose; comparatively, NAb (13%) had a rapidly positive transformation at four weeks. After booster stimulation, the positive proportion increased by 16‐ and 6‐times for IgG and NAb, respectively, on day 7. For IgG and IgM, the positive rate increased to 41% and 88% on day 14 after the second dose; after this point, the positive rate declined. During the visit period, the maximum seroconversion of NAb was detected after 28 days after the second dose. Except for one case, all other samples (58/59) experienced positive transformation.

Antibody levels changed over both vaccination time and procedure (Figure 3D,E). Within a four‐week observation period, both IgM and IgG levels were elevated on day 14 after each vaccination, followed by a decrease on the following weeks. Interestingly and different from the former two, NAb levels were continuously increased with increased vaccination time and again after booster stimulation. Compared to the pre‐inoculation base value of 0.50 AU/ml (95%CI: 0.47–0.55 AU/ml), NAb levels increased by five‐fold (2.49 AU/ml, 95%CI: 0.06–3.37 AU/ml) at day 28 after the first inoculation. Comparatively, levels were enhanced by 27‐fold (13.55 AU/ml) at the same visit point after the booster dose. There were no significant differences in NAb levels at either 14 or 28 days after the second vaccine (*p* > 0.05). However, it was notable that NAb levels were significantly increased in 53% participants and declined in 47% from 14 days to 28 days after the second dose (Figure 3G,F). At three months and regardless of whether assessing the group with increased or decreased levels, NAb levels were significantly reduced to 6.03 AU/ml when compared with the level observed on day 28 after completing the vaccination procedure. Six months later, the NAb levels were only 25% (the mean from 13.55 AU/ml to 3.34 AU/ml) of their peak levels.

### 3.4 SARS‐CoV‐2 IgM, IgG, and NAb changes in patients recovered from COVID‐19

**FIGURE 4:**
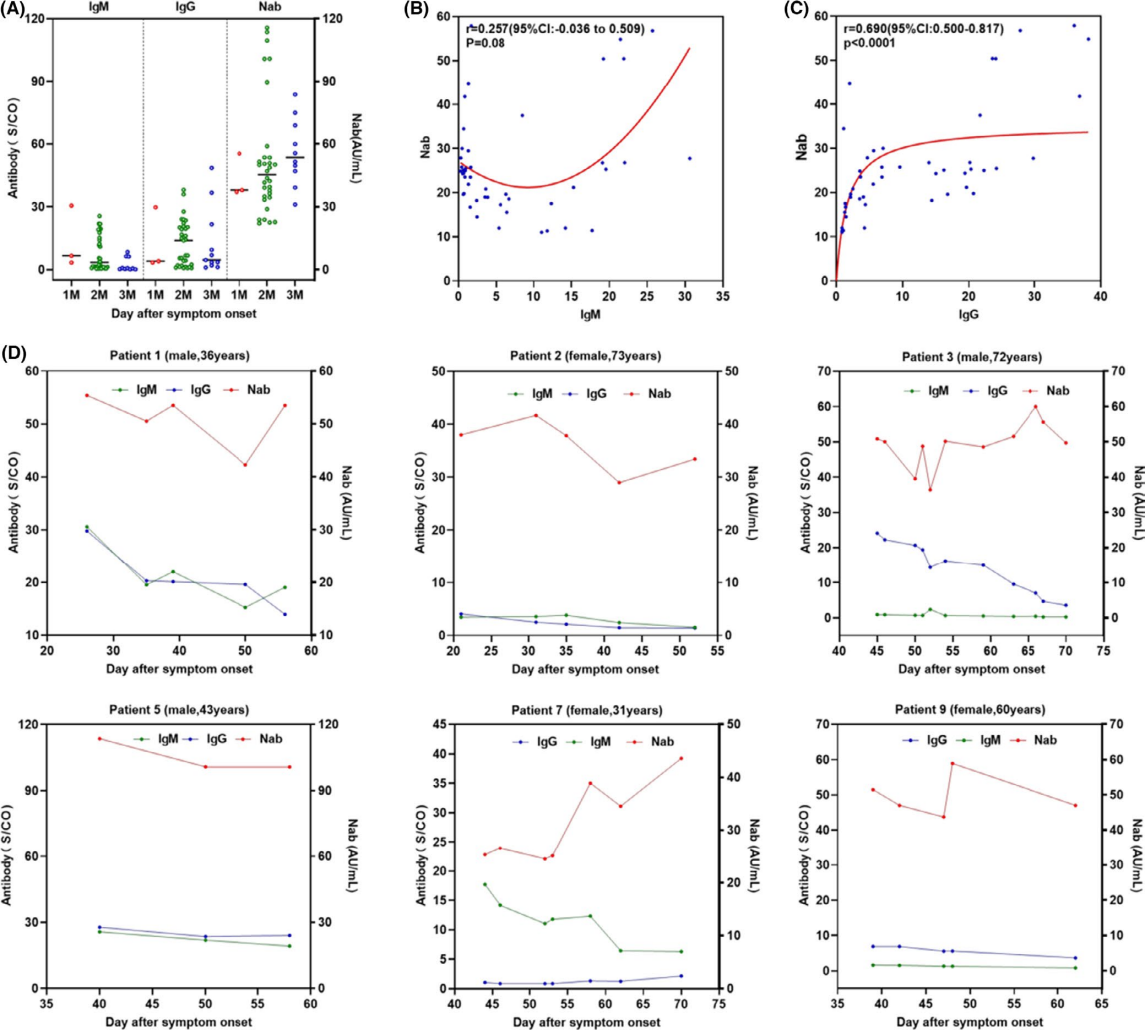
Antibody responses against SARS‐CoV‐2 over time. (A) The levels of antibody in COVID‐19 rehabilitation clients were changed with the time of rehabilitation. 1M, 2M, and 3M represent <30 days, 30–60 days, and >60 days, respectively. (B) Correlations between NAb levels and IgM levels from serum samples collected 10 COVID‐19 convalescent patients. (C) Correlations between Nab levels and IgG levels from serum samples collected 10 COVID‐19 convalescents. (D–I) Dynamic profile of IgM and IgG antibodies in representative six COVID‐19 patients after symptom onset

Surrogate antibody assays were also used in patients who had recovered from COVID‐19 and the IgM, IgG, and NAb results are shown in Figure 4.Seropositivity rates for IgM, IgG, and NAb were 70%, 93%, and 100%, respectively. For antibody levels, IgM dropped from 13.6 S/CO at 30 days post rehabilitation to 2.5 S/CO at 60 days, but the change was not significant (*p* > .05). Comparatively, IgG increased early, but declined later from 30 to 60 days post‐convalescence. NAb levels showed an increasing trend from 43.5 to 56.1 AU/ml within 60 days. Vaccine‐induced NAb levels were 7.41 AU/ml (95%CI: 5.02–9.8 AU/ml, 60 days), which was 0.14 times that of convalescent patients (52.47 AU/ml, 95%CI:45.18–59.76 AU/ml, 30–60 days). In comparison with SARS‐CoV‐2 IgM, the levels of SARS‐CoV‐2 IgG in convalescent patients had a stronger correlation (r=0.69, *p* < 0.0001) with that of NAb (Figure 4B,C). A follow‐up study was performed on six convalescent patients (Figure 4D), in which SARS‐CoV‐2 IgM and IgG levels in all six patients had decreased. NAb in recovered individuals were higher than both IgM and IgG; over time, four out of the six patients exhibited a slight decline.

## 4 DISCUSSION

NAb is a key factor in the prevention of SARS‐CoV‐2, and a neutralization assay is useful for high‐throughput evaluation of the effects of COVID‐19 vaccine as well as for identification of convalescent plasma for use in COVID‐19 treatment. The antibodies induced by an inactivated vaccine are similar to those found in the serum of convalescent patients. Previously published studies indicated that NAb levels were an important indicator for immunity protection against SARS‐CoV‐2.^14^, ^15^, ^16^ Both sera produced immunoreactivity, with the predominant antibodies targeted at either RBD or spike protein(S) and which induced a strong neutralization reaction.

Here, the clinical performances of three CLIA assays and two ELISA assays aimed at the RBD protein as well as the specific SARS‐CoV‐2 IgM and IgG proteins were evaluated and compared with cVNT. We observed good concordance above 0.78 between cVNT and the five surrogate NAb assays. As a surrogate assay, these rapid methods had great specificity at 100% and sensitivity, the latter of which ranged from 84.0% to 100%. It reported 95%–100% sensitivity and 99.93% specificity for cPass kit, which was verified with convalescent serum by cVNT and was ultimately authorized by the FDA for NAb testing.^11^ In comparison with cPass, the moderate agreement (kappa, 0.55–0.70) of the diagnostic result was obtained by four surrogate assays, the value was similar with a previous study (kappa, 0.61–0.74).^17^ Based on cELISA, clinical performance was similar with the approved cPass kit. Yaneng showed a slightly better correlation with cPass relative to the other three surrogate assays, since ELISA targeted the RBD antibody which was applied to both.

Similar to cVNT, all of above rapid methods were designed such that the antibodies competed with ACE2 to combing RBD, thus resulting in good clinical performance.^18^, ^19^, ^20^ We detected a moderate correlation (0.44–0.72) with cVNT for the NAb titer, indicating that the presences of unknown NAbs were likely generated against other viral proteins and non‐RBD regions of SARS‐CoV‐2. Except for the efficiency of the alpha strain (B.1.1.7) with serum NAb, the beta strain (B.1.351) was also detected in three vaccinated serum samples. A published study on BNT162b2 and ChAdOx1 vaccines showed effectiveness against the SARS‐CoV‐2 delta variant.^21^ In this study and compared to the average level (13.55 AU/ml), individual responses to the variant strain had higher NAb levels (39.10, 42.94, and 43.21 AU/ml). Collectively, these results demonstrated that surrogate assays may be valuable for evaluating vaccine efficiency, with high NAb levels against the emerging variants of SARS‐CoV‐2. In comparison with cVNT performed in a BSL‐3 laboratory, these surrogate assays could be conducted on an automatic platform, which would reduce exposure risk to the live virus.

The inactive COVID‐19 vaccine induced a humoral response in all participants after the two‐dose vaccination procedure was finished. In particular, seropositivity rapidly increased from 13% to 97% after the booster dose. Moreover, NAb levels were elevated from 2.48 AU/ml at day 28 after the first dose to 24.5 AU/ml at day 28 after the second dose. These results were similar with previous findings.^22^, ^23^ Interestingly, the mRNA vaccine induced a NAb response after the first dose in less than half of individuals.^7^ The inactivated BBIBP‐CorV and CoronaVac vaccines have been reported to boost immunization seropositivities between 95% and 100%.^24^, ^25^ The second dose was shown to be necessary for enhancing antibody titers and building a defense against the SARS‐CoV‐2 virus. Except for one participant, all others produced a robust humoral immunity after exposure to the inactive vaccine during the four‐month observation period. Half of vaccine recipients had NAb levels that declined at 28 days post‐vaccine, compared with those at 14 days after receiving the second dose. There was no significant difference in either age or gender between the two groups. Referring to the NAb response in infectious individuals, a proportion remained at a high NAb titer and decreased from 52.8% to 27.6% at one and two months after COVID‐19 onset.^26^ A bigger question remained regarding how long antibody levels lasted. Within the four‐month monitoring period and although NAb levels dropped over time, the seropositive rate of NAb remained at 84%. However, at six months post‐vaccination, these levels had decreased on average to 25% of their peak levels. A previous study reported there is a substantial decrease in the titer of spike protein of SARS‐CoV‐2 in humans after 200 days of BioNTech/Pfizer's BNT162b2 COVID‐19 vaccination.^27^ Besides, this decline was similar to the observed in other mRNA vaccine studies.^28^, ^29^ Future studies will be required to follow‐up with whether antibody levels will continue to decline or plateau at a lower level. Aiming to maintain effectiveness against the severity of COVID‐19, enough humoral and cellular immune response after vaccination was required. Hence, the booster dose may be needed at six months post‐vaccination, especially for individuals with low NAb level. The studies on BNT162b2 and mRNA‐1273 vaccines also suggested a booster with an interval of six to eight months after the immunization procedure.^30^, ^31^ A remaining question was whether routine analysis with the specific IgM or IgG to SARS‐CoV‐2 could be a diagnostic marker for the effectiveness of the vaccine used. To answer this, an increase in the IgA and IgG index has been shown to indicate the generated immune response to the mRNA‐based vaccine. However, the seroconversion was not reported.^32^ Here, the IgM and IgG titers rapidly increased, but decreased within a two‐week interval after each vaccination. To assess the diagnostic capacity of IgM and IgG, we used a given cutoff (1 S/CO) which resulted in poor specificities of 0.32 and 0.84, respectively. Given this, NAb was defined as a satisfactory indicator for the effective evaluation and immune response of COVID‐19 vaccines.

The NAb titer was significantly correlated with IgG level (r, 0.410) in infectious samples at three weeks after symptom onset.^33^ Our results showed a good correlation coefficient at 0.69 in convalescents. Moreover, NAb remained at a long‐term positive rate and at a higher titer relative to other antibodies after discharge from hospital. This was likely related to age, sex, disease severity, and/or hospitalization.^34^ It was also observed that vaccine‐induced NAb levels were lower than those generated from COVID‐19 convalescent plasma. This finding was different from that observed in the study on the KCONVAC vaccine, which showed NAb levels 2.65 times greater than recovered patients.^35^ This may be due to a combination of factors, including the great replicability rate of SARS‐CoV‐2, upregulated expression of encoding proteins, and generation of a strong and robust immune response. The humoral immune responses with sustained NAb titer were essential factors to cellular immunity and also contributed to protecting against SARS‐CoV‐2 infection and illness and will be required for future study. NAb monitoring in a follow‐up cohort was limited to targeting at the RBD protein and may not represent the actual situation (e.g., NAb against unknown or non‐RBD proteins).^36^

In summary, the surrogate assay was provided as a proxy for the large‐scale and dynamic monitoring of NAb titers inCOVID‐19 vaccination and infection cohorts. Inactive COVID‐19 vaccines generated a gradually emerging humoral immunity response along with the inoculation procedure; critically, antibodies persisted at high titers for at least six months.

## CONFLICT OF INTERESTS

The authors declare there are no conflicts of interest to disclose.

## AUTHOR CONTRIBUTIONS

All authors were involved in the analysis and interpretation of data as well as drafting the manuscript or revising it critically for important intellectual content. Xiuming Zhang, Xiaowen Dou, and Ruiwei Jiang made substantial contributions to the conception and design of the study and designed the experiments. Enyun Wang performed the serological assay experiments and the data collection. Dan Xiong and Min Li data analysis, statistical analysis and wrote the article. All authors read and approved the final manuscript.

## Supporting information

Supplementary Material

## Data Availability

The data that support the findings of this study are available from the corresponding author upon reasonable request.
