# Supplementary material for: Dynamic observation of SARS‐CoV‐2 IgM, IgG, and neutralizing antibodies in the development of population immunity through COVID‐19 vaccination
Source: J Clin Lab Anal. 2022 Mar 2;36(4):e24325. doi: 10.1002/jcla.24325 (PMC8993648; doi:10.1002/jcla.24325)
Supplement: Supplementary file 1 — Supplementary Material [file JCLA-36-e24325-s001.docx]

**D****y****n****amic observation of****SARS-CoV-2 IgM, IgG, and neutralizing antibodies in the development of population immunity throughCOVID-19 vaccination**

Ruiwei Jiang^1, 3#^, Xiaowen Dou^2#^, Min Li^1,2^, Enyun Wang^3^, JiwenHu^3^, Dan Xiong^2,3^, Xiuming Zhang^1, 2, 3^*

^1^Anhui University of Science and Technology, Huainan, 232001, China;

^2^Medical Laboratory of the Third affiliated hospital of Shenzhen university, Shenzhen, 518001, China;

^3^Medical Laboratory of Shenzhen Luohu Hospital Group, Shenzhen Luohu People’s Hospital, Shenzhen, 518001, China;

^#^ Ruiwei Jiang and Xiaowen Dou the author works equally.

*Correspondence: Xiuming Zhang,[zhangxiuming0760@163.com](mailto:zhangxiuming0760@163.com)

TABLE S1.Baseline characteristics of participants

| Characteristics | Vaccine recipients | Convalescents |
| --- | --- | --- |
| Overall | 164(100%) | 10(100%) |
| Age,years |  |  |
| 20-29 | 28(17%) |  |
| 30-39 | 37(23%) | 2(20%) |
| 40-49 | 51(31%) | 2(20%) |
| >50 | 47(29%) | 6(60%) |
| Sex |  |  |
| Male | 76(46%) | 4(40%) |
| Female | 88(54%) | 6(60%) |
| Underlying disease |  |  |
| Yes | 5(3%) | 4(60%) |
| No | 159(97%) | 6(40%) |

*：Seven participants were mainly hypertension, allergic rhinitis,bronchiectasis and tuberculosis


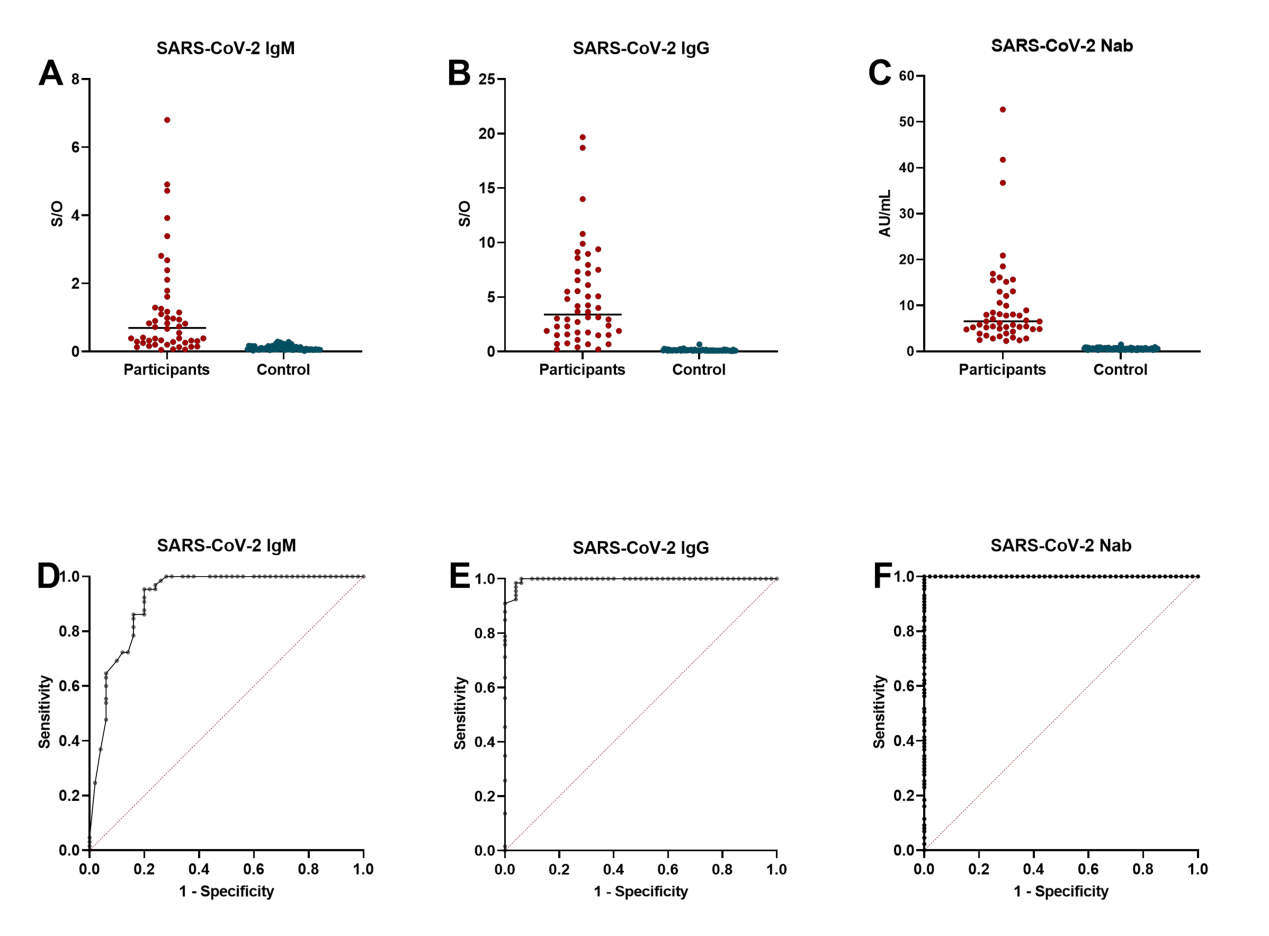


FIGURE S1. (A-C)Scatter plot of SARS-CoV-2 IgM, IgG and Nab between COVID-19 vaccination cohort (*n*=50) and control group (*n*=50) without vaccination. COVID-19 vaccination cohort was identified with Nab titer above 20 by cVNT. Receiver operating characteristic (ROC) curves for each antibody was drawn.


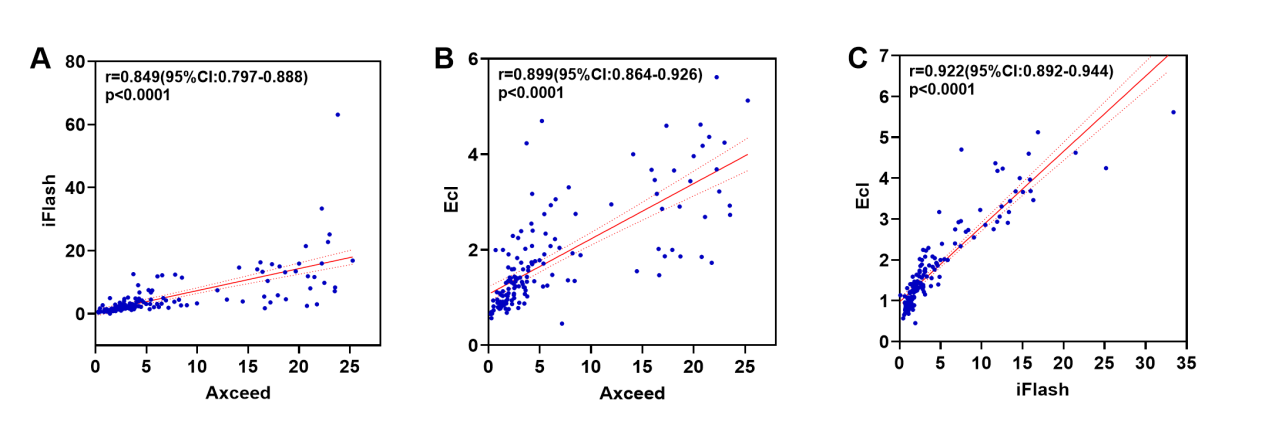


FIGURE S2. Comparison of correlation of three chemiluminescence methods.
